# Supplementary material for: Distinct transcriptional roles for Histone H3-K56 acetylation during the cell cycle in Yeast
Source: Nat Commun. 2019 Sep 26;10:4372. doi: 10.1038/s41467-019-12400-5 (PMC6763489; doi:10.1038/s41467-019-12400-5)
Supplement: Supplementary file 1 — Supplementary Information [file 41467_2019_12400_MOESM1_ESM.pdf]

SUPPLEMENTARY INFORMATION

Distinct transcriptional roles for Histone H3-K56 acetylation during the cell cycle in Yeast

Topal *et al*.

Supplementary Figure 1

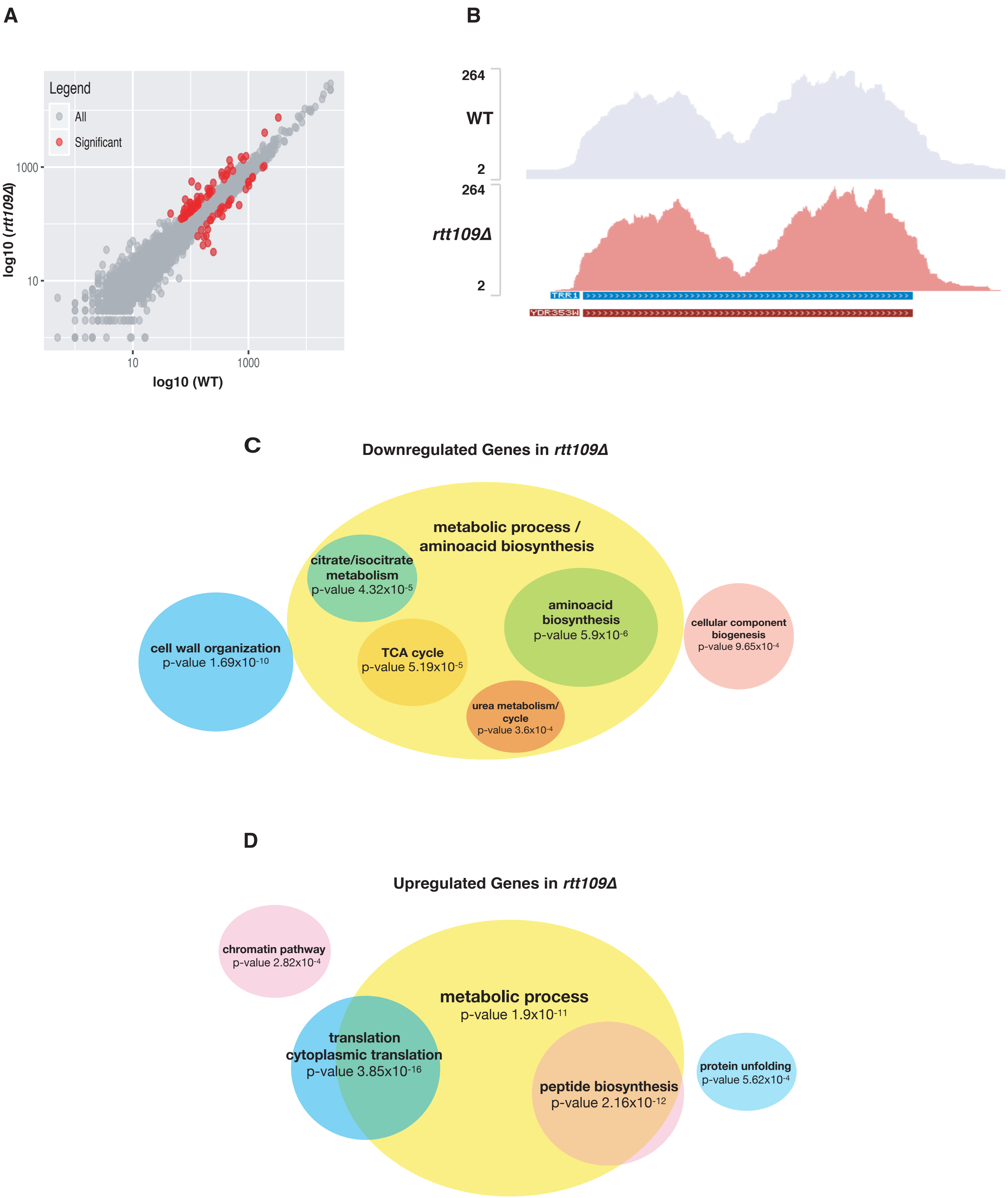

Supplementary Figure 1, RNA-seq data.

(A) Significance plot showing nascent coding transcript levels between wild type and *rtt109Δ* by RNA-seq. Significantly changed (≥1.5 FC, FDR ≤ 0.05) genes (red) and non-significant genes (gray). Data were normalized according to spike-in numbers. **(B)** Genome browser view of an example gene (*YDR353W*) between wild type (gray) and *rtt109Δ* (red). **(C,D)** GO-Term analysis for down-regulated or up-regulated genes from **(A)**. Significance and p-values were calculated by using Mann-Whitney U-test.

Supplementary Figure 2

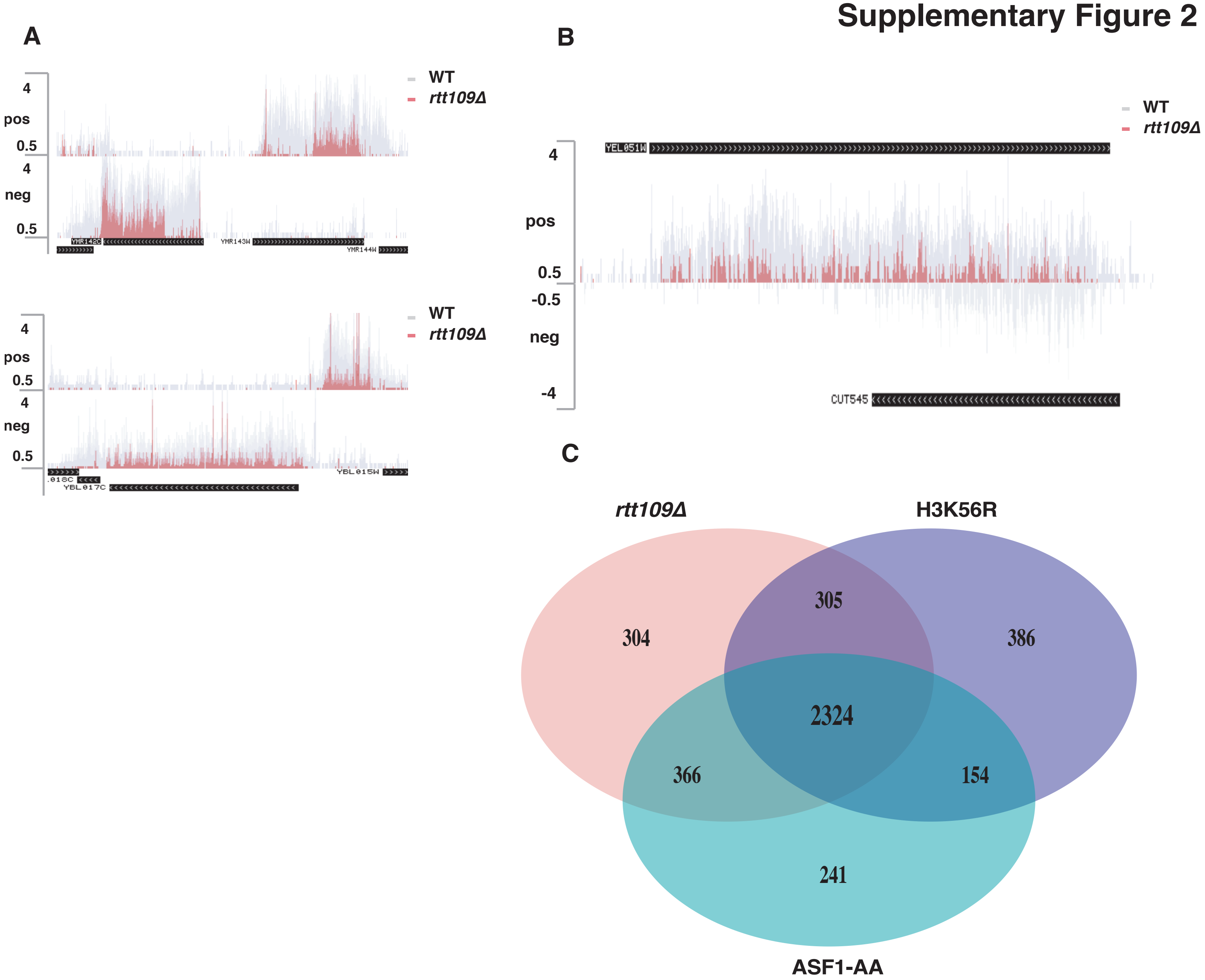

number of genes downregulated > 1.5-fold compared to WT

Supplementary Figure 2, related to Figures 1 and 2.

(A-B) Genome browser views of two example protein-coding genes **(A)**, and of a CUT, CUT545 **(B)** between wild type (gray) and *rtt109Δ* (red). **(C)** Venn diagram showing correlation in numbers of down-regulated genes (≥ 1.5 FC, FDR ≤ 0.05) between *rtt109Δ*, Asf1-AA and H3K56R.

Supplementary Figure 3

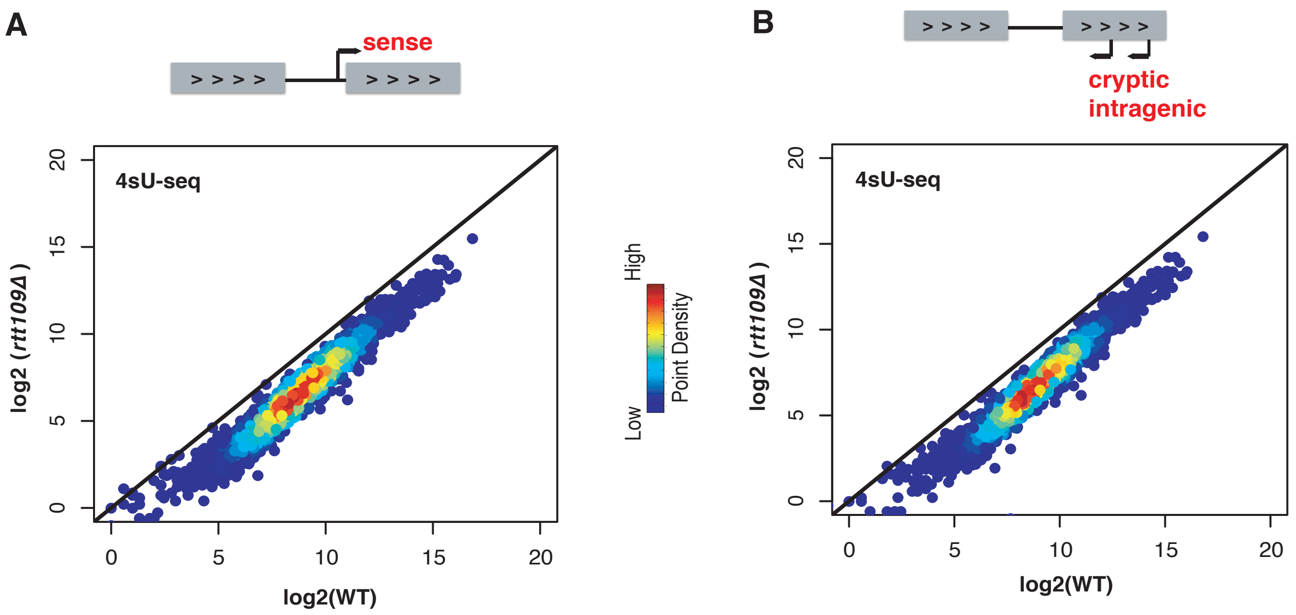

Supplementary Figure 3, related to Figure 3.

(A-B) Scatterplots showing nascent coding transcript levels **(A)**, and cryptic intragenic transcript levels **(B)** between wild type and *rtt109Δ* by 4sU-seq. Data were normalized by spike-in numbers.

Supplementary Figure 4

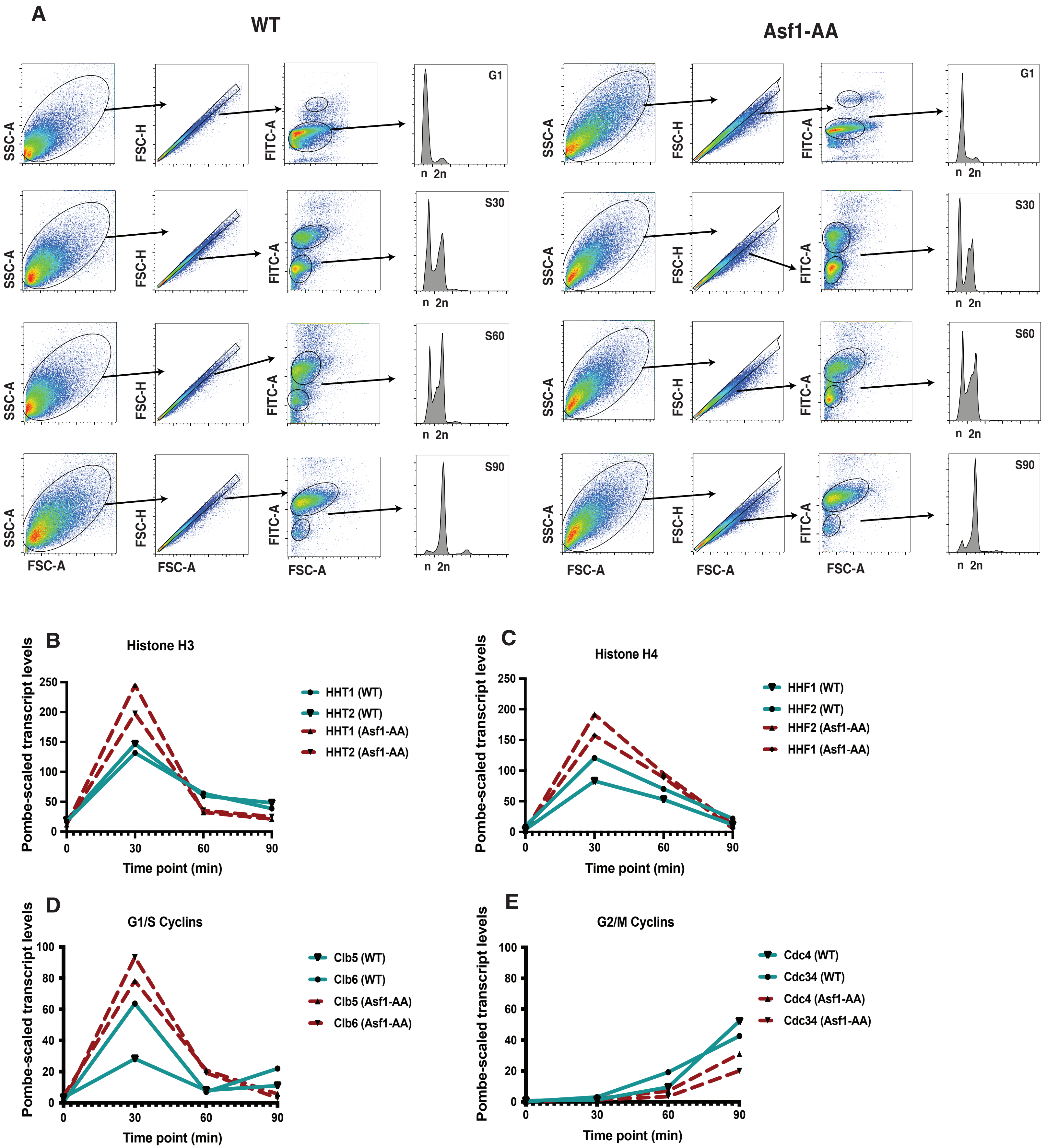

Supplementary Figure 4, related to Figure 4.

(A) FACS analysis and gating strategies used for G1-arrested samples (α-factor), early S-phase (+30 min), late S-phase (+60 min) and G2/M phase (+90 min) for WT (left) and Asf1-AA (right). After gating on SC-FSC cytoplots, 90-95% of cells were used in the next gating (FSC-H vs. FSC-A). 75-85% of these cells were single cells (determined by FSC-H vs. FSC-A) that were used to determine DNA content under FITC channel. **(B-E)** Plots showing normalized transcript levels of cell-cycle regulated genes at different time points (G1, S30, S60 and S90) for Histone H3 genes **(B)**, Histone H4 genes **(C)**, G1/S cyclins **(D)**, and G2/M cyclins **(E)** for wild type and Asf1-AA.

Supplementary Figure 5

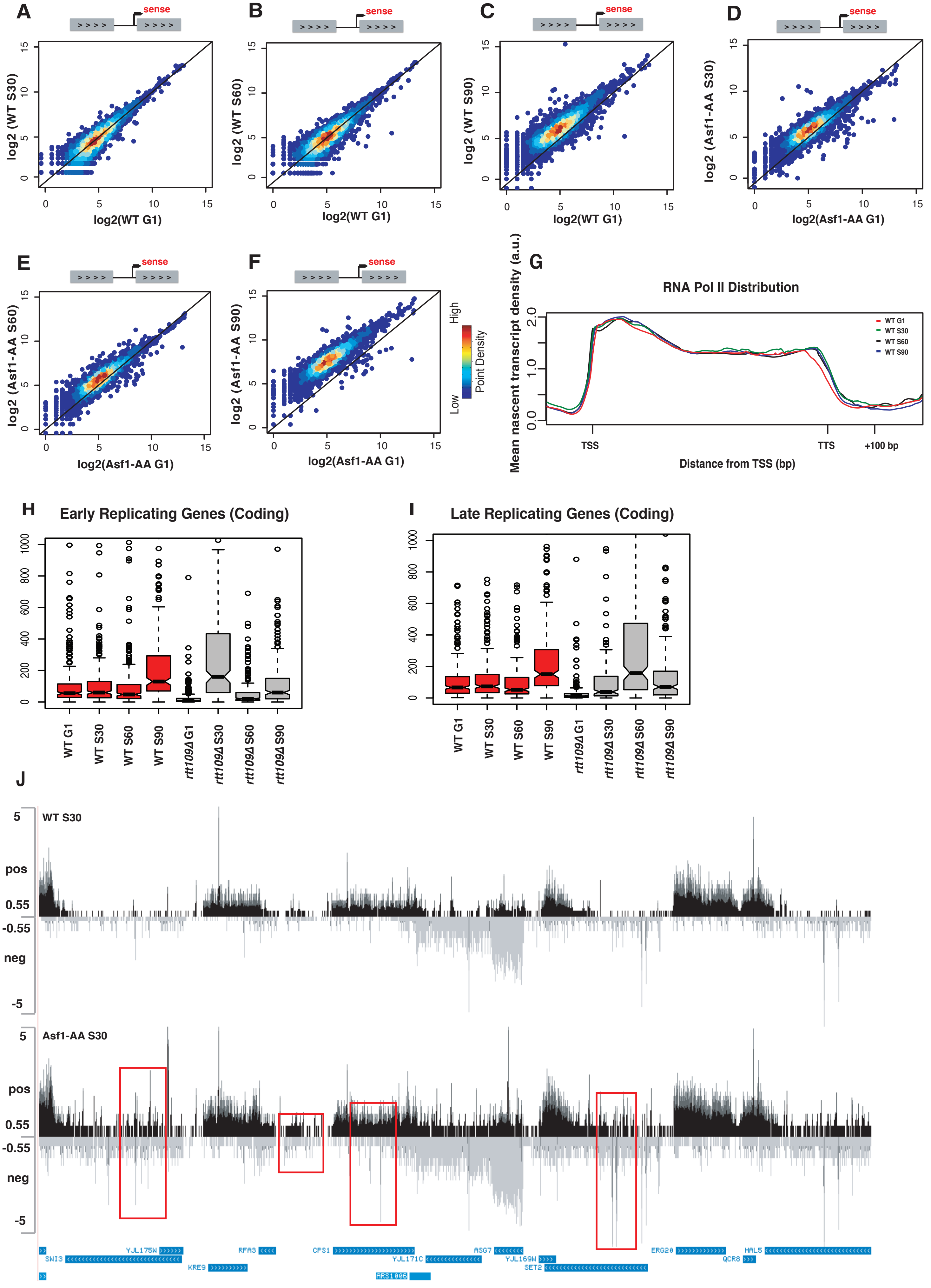

Supplementary Figure 5, related to Figure 4.

(A-C) Scatterplots showing nascent coding transcript levels between G1 and S30 time point **(A)**, S60 time point **(B)**, and S90 time point **(C)** in wild type cells; levels between G1 and S30 time point **(D)**, S60 time point **(E)**, and S90 time point **(F)** in Asf1-AA cells. **(G)** Metagene plot showing RNA Pol II distribution throughout the gene body from TSS to TTS (including 100 bp upstream and 200 bp downstream of TTS) fitted into 500 bp. Plots are shown as wild type G1 (red), S30 time point (green), S60 time point (black) and S90 time point (blue). The mean nascent transcript levels are normalized according to both spike-in numbers and each gene's individual expression level. **(H, I)** Boxplots showing nascent transcript levels at different time points (G1, S30, S60 and S90) between wild type (red) and *rtt109Δ* (gray) for early replicating genes **(H)** or late replicating genes **(I)** for coding regions. The lateral lines in the boxes represent the median, and separate upper and lower quartiles. The vertical lines represent the highest and lowest data points. Significance and p-values were calculated by using Mann-Whitney U-test. Error bars represent standard deviation. **(J)** Genome browser view of several genes around an early replication origin (ARS1006). Notice immediate transcriptional bursts (as indicated in red boxes) in Asf1-AA in S30 time point. Data were normalized according to spike-in numbers. A.U. arbitrary unit.

Supplementary Figure 6

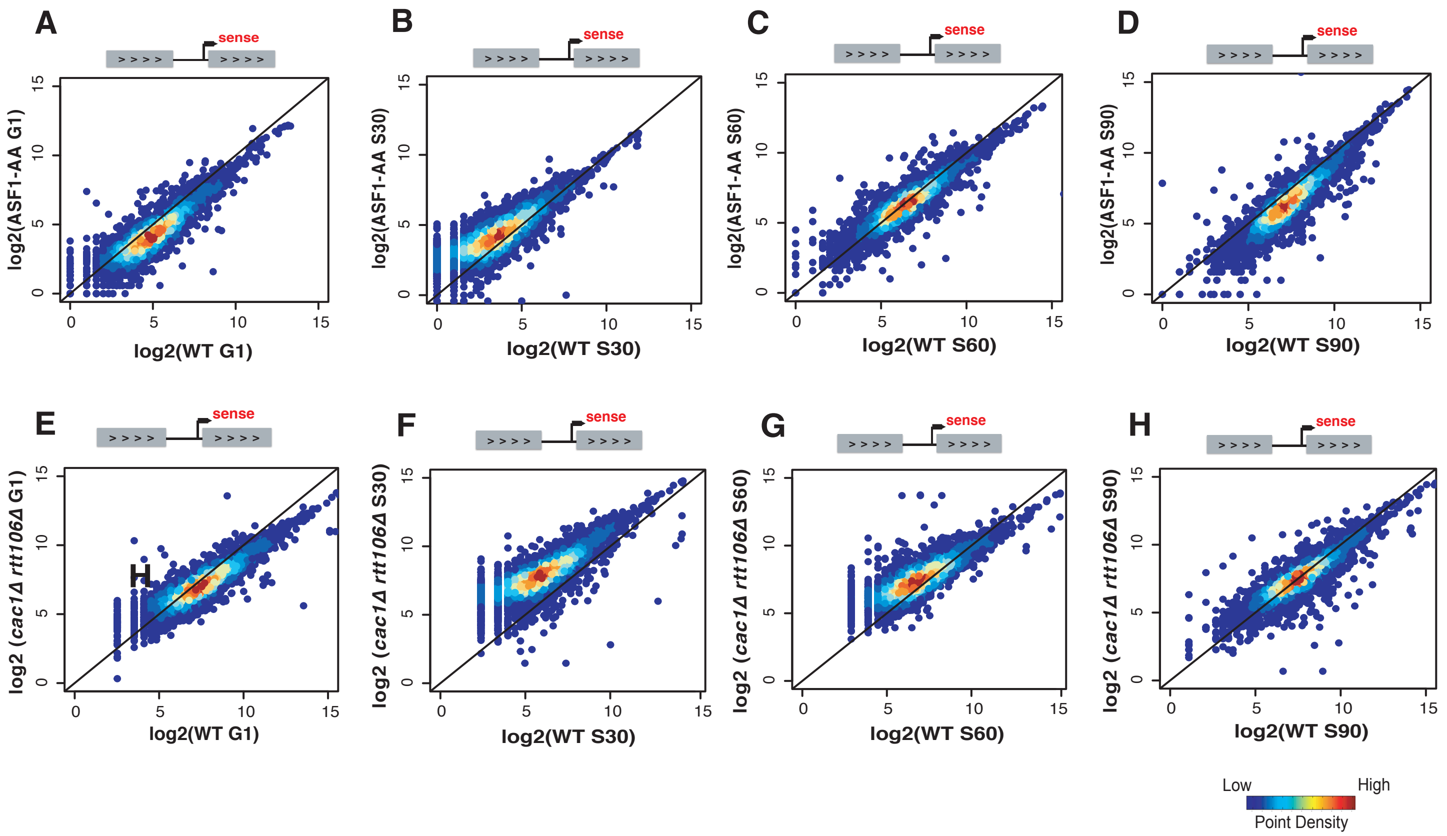

Supplementary Figure 6, related to Figure 4 and Figure 5.

(A-H) Scatterplots showing nascent coding transcript levels between wild-type and Asf1-AA for G1 **(A)**, S30 **(B)**, S60 **(C)**, and S90 **(D)**; between wild-type and *cac1Δ rtt106Δ* for G1 **(E)**, S30 **(F)**, S60 **(G)**, and S90 **(H)**. Data were normalized according to spike-in numbers.
