# Supplementary material for: Distinct transcriptional roles for Histone H3-K56 acetylation during the cell cycle in Yeast
Source: Nat Commun. 2019 Sep 26;10:4372. doi: 10.1038/s41467-019-12400-5 (PMC6763489; doi:10.1038/s41467-019-12400-5)
Supplement: Supplementary file 3 — Description of Additional Supplementary Files [file 41467_2019_12400_MOESM3_ESM.pdf]

Description of Additional Supplementary Files

File Name: Supplementary Data 1
Description: The list of early replicating genes

File Name: Supplementary Data 2
Description: The list of late replicating genes

File Name: Supplementary Data 3
Description: The list of cell cycle regulated genes
