# Supplementary material for: Distinct transcriptional roles for Histone H3-K56 acetylation during the cell cycle in Yeast
Source: Nat Commun. 2019 Sep 26;10:4372. doi: 10.1038/s41467-019-12400-5 (PMC6763489; doi:10.1038/s41467-019-12400-5)
Supplement: Supplementary file 7 — Reporting Summary [file 41467_2019_12400_MOESM7_ESM.pdf]

nature research

Corresponding author(s): Craig L Peterson

Last updated by author(s): Aug 27, 2019

Reporting Summary

Nature Research wishes to improve the reproducibility of the work that we publish. This form provides structure for consistency and transparency in reporting. For further information on Nature Research policies, see Authors & Referees and the Editorial Policy Checklist.

Statistics

For all statistical analyses, confirm that the following items are present in the figure legend, table legend, main text, or Methods section.

n/a	Confirmed	
☐	☒	The exact sample size (*n*) for each experimental group/condition, given as a discrete number and unit of measurement
☐	☒	A statement on whether measurements were taken from distinct samples or whether the same sample was measured repeatedly
☐	☒	The statistical test(s) used AND whether they are one- or two-sided<br>*Only common tests should be described solely by name; describe more complex techniques in the Methods section.*
☒	☐	A description of all covariates tested
☒	☐	A description of any assumptions or corrections, such as tests of normality and adjustment for multiple comparisons
☒	☐	A full description of the statistical parameters including central tendency (e.g. means) or other basic estimates (e.g. regression coefficient) AND variation (e.g. standard deviation) or associated estimates of uncertainty (e.g. confidence intervals)
☒	☐	For null hypothesis testing, the test statistic (e.g. *F*, *t*, *r*) with confidence intervals, effect sizes, degrees of freedom and *P* value noted<br>*Give P values as exact values whenever suitable.*
☒	☐	For Bayesian analysis, information on the choice of priors and Markov chain Monte Carlo settings
☒	☐	For hierarchical and complex designs, identification of the appropriate level for tests and full reporting of outcomes
☒	☐	Estimates of effect sizes (e.g. Cohen's *d*, Pearson's *r*), indicating how they were calculated

Our web collection on statistics for biologists contains articles on many of the points above.

Software and code

Policy information about availability of computer code

Data collection	For the RNA sequencing analysis, NET-seq, RNA-seq, TT-seq and 4sU-seq libraries were sequenced using Illumina NextSeq 550 (75 bp single-end for NET-seq and 150 bp paired-end for RNA-seq, TT-seq and 4sU-seq).
Data analysis	For NET-seq analysis, reads were processed and aligned using Galaxy web platform (Afgan et al., 2018). The adapter sequence was (ATCTCGTATGCCGTCTTCTGCTTG) removed and the random hexamer sequence was removed from the 5' end. The 3' ends of the reads were then trimmed for quality using FASTQ Quality Timmer by sliding window (Blankenberg et al., 2010) with a window size of 10 and a step size of 5. The reads were trimmed until the aggregate score was ≥ 21. Reads were first aligned using Bowtie2 (Langmead and Salzberg, 2012; Langmead et al., 2009) to a combined FASTA file of S. cerevisiae and S. pombe rRNA, tRNA, and RDN sequences to remove contaminating reads. Reads were then aligned to a combined version of the S. cerevisiae genome (SacCer3, SGD) and the S. pombe genome (ASM294v.2, PomBase) with TopHat2 (Kim et al., 2013), allowing up to three mismatches. The reads were separated by their respective genomes with SAMtools (Li et al., 2009), and only uniquely mapped reads were used for further analyses. Libraries were normalized by scaling the uniquely mapped S. pombe reads to 100,000 reads. This scaling factor was then used to scale the uniquely mapped S. cerevisiae reads. To account for differences between sequencing run depth for various NextSeq runs, the pombe-scaled WT S. cerevisiae read counts were then scaled to 1 M reads, and this additional scaling factor was included to scale the sample reads. Finally, only the 5' end of the sequencing read, which corresponds to the 3' end of the nascent RNA was recorded and used for downstream analyses. TSS and TTS annotation was obtained from (Xu et al., 2009). Read counts for genes and non-coding regions were obtained by summing normalized base pair reads over the region of interest. For average profiles, BAM files of biological replicates were merged and processed as above, and only genes longer than 500 bp were analyzed. Genes were scaled to 500 bp, and samples were scored in 1 bp bins using the deepTools program (Ramirez et al., 2016). Reads were analyzed as in (Harlen et al., 2016). To calculate 5' to 3' ratios, the sum of reads from 1-250 bp from the TSS were divided by the sum of reads 250 bp upstream of the TTS to the TTS. For RNA-seq, TT-seq and 4sU-seq analyses, FASTQ files from paired end libraries were collapsed by barcode and the Illumina adapter sequence was trimmed from the 3' end. Files were uploaded and analyzed using the Galaxy web platform (Afgan et al., 2018). Reads were first aligned using Bowtie2 (Langmead and Salzberg, 2012; Langmead et al., 2009) to S. cerevisiae rRNA, tRNA, and RDN sequences to remove contaminating reads. Reads were then aligned to a combined version of the S. cerevisiae genome (SacCer3, SGD) and a list of ERCC spike-ins with TopHat2 (Kim et al., 2013) allowing up to two mismatches. The reads were separated by their respective genomes with SAMtools

(Li et al., 2009), and only uniquely mapped reads were used for further analyses. For visualization in USCS genome browser, libraries were normalized by ERCC spike-in numbers. To compare RNA expression between samples, HTseq 0.9.1 (Anders et al., 2015) was used to count the number of reads that aligned to each annotated gene. The annotation file for the S. cerevisiae genome was generated from the Xu et al. dataset (Xu et al., 2009). Differential expression analysis was performed using edgeR (Robinson et al., 2010) or in excel using the R qvalue package.

For manuscripts utilizing custom algorithms or software that are central to the research but not yet described in published literature, software must be made available to editors/reviewers. We strongly encourage code deposition in a community repository (e.g. GitHub). See the Nature Research guidelines for submitting code & software for further information.

Data

Policy information about availability of data

All manuscripts must include a data availability statement. This statement should provide the following information, where applicable:
- Accession codes, unique identifiers, or web links for publicly available datasets
- A list of figures that have associated raw data
- A description of any restrictions on data availability

The accession number for the raw and processed NET-seq, RNA-seq, TT-seq and 4sU-seq data is GEO:GSE125843. The accession numbers for the NChAP data are GEO:GSE74090 and GEO:GSE126686.

Field-specific reporting

Please select the one below that is the best fit for your research. If you are not sure, read the appropriate sections before making your selection.

☒ Life sciences ☐ Behavioural & social sciences ☐ Ecological, evolutionary & environmental sciences

For a reference copy of the document with all sections, see nature.com/documents/nr-reporting-summary-flat.pdf

Life sciences study design

All studies must disclose on these points even when the disclosure is negative.

Sample size	No statistical method was used to predetermine sample size.
Data exclusions	No data were excluded from analyses.
Replication	The reproducibility for all sequencing data and analyses was confirmed by two independent experiments.
Randomization	This was not relevant to our study.
Blinding	This was not relevant to our study.

Reporting for specific materials, systems and methods

We require information from authors about some types of materials, experimental systems and methods used in many studies. Here, indicate whether each material, system or method listed is relevant to your study. If you are not sure if a list item applies to your research, read the appropriate section before selecting a response.

Materials & experimental systems

n/a	Involved in the study	
☐	☒	Antibodies
☒	☐	Eukaryotic cell lines
☒	☐	Palaeontology
☒	☐	Animals and other organisms
☒	☐	Human research participants
☒	☐	Clinical data

Methods

n/a	Involved in the study	
☒	☐	ChIP-seq
☐	☒	Flow cytometry
☒	☐	MRI-based neuroimaging

Antibodies

Antibodies used	Anti-FLAG M2 affinity gel (Millipore Sigma Cat#A2220)
Validation	The commercial antibody was validated by their producer.

Flow Cytometry

Plots

Confirm that:

☒ The axis labels state the marker and fluorochrome used (e.g. CD4-FITC).

☒ The axis scales are clearly visible. Include numbers along axes only for bottom left plot of group (a 'group' is an analysis of identical markers).

☒ All plots are contour plots with outliers or pseudocolor plots.

☒ A numerical value for number of cells or percentage (with statistics) is provided.

Methodology

Sample preparation	Samples were collected at certain time points at an OD600 of 0.6-0.8. Following spinning down cells, they were resuspended in 70% ethanol for overnight incubation at 4C. Following day, cells were sonicated once at setting 3 for 5 seconds (Sonic Dismembrator 550, Fisher Scientific). After sequential resuspension in distilled water and 50 mM Tris (pH 8.0), cells were incubated in RNase A (10 mg/ml) at 37C for 3-4 hours. After resuspension in 50 mM Tris (pH 7.5), cells were incubated in Proteinase K (2 mg/ml) at 50C for 1 hour. Then, they were resuspended in FACS buffer (200 mM Tris-HCl (pH 7.5), 200 mM NaCl, 78 mM MgCl2). Cells were incubated with 1X Sytox Green before collecting in BD FACSDiva Software and analyzing in Flowjo v10.6.0.
Instrument	BD Biosciences LSRII
Software	BD FACSDiva Software was used for data collection and Flowjo v10.6.0 was used for data analysis.
Cell population abundance	~100,000 cells were initially collected and 70-85% of these were single cells. Since only one marker (Sytox Green) was used, purity criteria does not apply.
Gating strategy	Positive staining for Sytox Green (FITC) for cells were determined by using unstained cells. After gating on SSC-FSC cytoplot, FSC-H vs FSC-A was used to determine only single cells. FITC channel was used to assess the cell cycle.

☒ Tick this box to confirm that a figure exemplifying the gating strategy is provided in the Supplementary Information.
